# Transcriptomic analysis of human primary breast cancer identifies fatty acid oxidation as a target for metformin

**DOI:** 10.1038/s41416-019-0665-5

**Published:** 2019-12-10

**Authors:** Simon R. Lord, Jennifer M. Collins, Wei-Chen Cheng, Syed Haider, Simon Wigfield, Edoardo Gaude, Barbara A. Fielding, Katherine E. Pinnick, Ulrike Harjes, Ashvina Segaran, Pooja Jha, Gerald Hoefler, Michael N. Pollak, Alastair M. Thompson, Pankaj G. Roy, Ruth. English, Rosie F. Adams, Christian Frezza, Francesca M. Buffa, Fredrik Karpe, Adrian L. Harris

**Affiliations:** 1 Department of Oncology, University of Oxford, Churchill Hospital, Oxford, OX3 7LE UK; 2 Molecular Oncology Laboratories, Weatherall Institute of Molecular Medicine, University of Oxford, John Radcliffe Hospital, Oxford, OX3 9DS UK; 3 NIHR Oxford Biomedical Research Centre, Oxford University Hospitals NHS Foundation Trust, Oxford, UK; 4 Oxford Centre for Diabetes, Endocrinology and Metabolism, Radcliffe Department of Medicine, University of Oxford, Churchill Hospital, Oxford, OX3 7LE UK; 5 The Breast Cancer Now Toby Robins Research Centre, The Institute of Cancer Research, London, SW3 6JB UK; 6 MRC Cancer Unit, University of Cambridge, Hutchison/MRC Research Centre, Cambridge Biomedical Campus, Cambridge, CB2 0XZ UK; 7 Faculty of Health and Medical Sciences, University of Surrey, Guildford, Surrey GU2 7WG UK; 8 Institut für Pathologie, Medizinische Universität Graz, Auenbruggerplatz 25, 8036 Graz, Austria; 9 Department of Oncology, McGill University, Montreal, QC H3T 1E2 Canada; 10 Division of Surgical Oncology, Baylor College of Medicine, Houston, TX 77030 USA; 11 Breast Surgery Unit, Oxford University Hospitals NHS Foundation Trust, Churchill Hospital, Oxford, OX3 7LE UK; 12 Oxford Breast Imaging Centre, Oxford University Hospitals NHS Foundation Trust, Churchill Hospital, Oxford, OX3 7LE UK

**Keywords:** Cancer metabolism, Breast cancer, Target identification, Cancer genomics

## Abstract

**Background:**

Epidemiological studies suggest that metformin may reduce the incidence of cancer in patients with diabetes and multiple late phase clinical trials assessing the potential of repurposing this drug are underway. Transcriptomic profiling of tumour samples is an excellent tool to understand drug bioactivity, identify candidate biomarkers and assess for mechanisms of resistance to therapy.

**Methods:**

Thirty-six patients with untreated primary breast cancer were recruited to a window study and transcriptomic profiling of tumour samples carried out before and after metformin treatment.

**Results:**

Multiple genes that regulate fatty acid oxidation were upregulated at the transcriptomic level and there was a differential change in expression between two previously identified cohorts of patients with distinct metabolic responses. Increase in expression of a mitochondrial fatty oxidation gene composite signature correlated with change in a proliferation gene signature. In vitro assays showed that, in contrast to previous studies in models of normal cells, metformin reduces fatty acid oxidation with a subsequent accumulation of intracellular triglyceride, independent of AMPK activation.

**Conclusions:**

We propose that metformin at clinical doses targets fatty acid oxidation in cancer cells with implications for patient selection and drug combinations.

**Clinical Trial Registration:**

NCT01266486.

## Background

Epidemiological and retrospective clinical studies suggest a reduction in relative risk of cancer associated with the diabetes drug, metformin, and multiple phase 3 clinical trials are now underway to assess the potential of repurposing metformin as an anti-cancer therapy.^1^ However, metformin’s anti-cancer mechanism of action remains unclear. The canonical view is that metformin activates 5′ AMP-activated protein kinase (AMPK) in cancer cells leading to metabolic reprogramming and inducing a limit on utilisation of nutrient resources, subsequently halting proliferation.^2^ Activation of AMPK is thought to be secondary to inhibition of complex 1 of the mitochondrial respiratory chain^3^ and growing evidence suggests that metformin modulates mitochondrial metabolism at clinical doses.^4,5^ In a clinical pharmacodynamic study of 36 breast cancer patients we recently showed that metformin treatment leads to two distinct metabolic responses.^4^ Here, we describe further analysis of our patient cohort in which we identify that multiple genes regulating fatty acid oxidation (FAO) are upregulated at the transcriptomic level following metformin treatment. Additionally, increase in expression of a composite mitochondrial FAO gene expression profile correlated with change in a proliferation gene signature and this signature discriminated between the patient groups with differential metabolic responses. In vitro assays showed that, in contrast to previous studies in models of normal cells, metformin reduces fatty acid oxidation with a subsequent accumulation of intracellular triglyceride, independent of AMPK activation.

## Methods

### Clinical study design and patient selection

Patients with a new diagnosis of primary breast cancer were recruited in three UK centres. The study was prospectively approved by the NHS Oxfordshire Research Ethics Committee A and registered with the ClinicalTrials.gov identifier: NCT01266486. Metformin was given in the Glucophage XR™ formulation in an escalating dose once daily for a minimum of 13 days and a maximum of 21 days (500 mg for days 1**–**3, 1000 mg for days 4**–**6 and 1500 mg thereafter). The day prior to commencing metformin a core biopsy was taken under ultrasound guidance from the periphery of the primary tumour and a second biopsy using the same approach after 13–21 days of metformin treatment as above. Within 1 min of this procedure the biopsy material was snap frozen in liquid nitrogen prior to storage at −80 °C. Please see Fig. S1A and Lord et al., Cell Metabolism for further details.^4^

### Bioinformatic analysis and statistical methods

Next-generation sequencing of ‘Poly (A) targeted’ mRNA was carried out for the clinical biopsy samples taken pre and post-metformin. The fold change of normalised expression level, FPKM (Fragments Per Kilobase of transcript per Million mapped reads), for each gene was then estimated from those aligned reads using Cuffdiff 2.2.1. Non-parametric rank product (R project v3.3.1) was used to prioritise the genes with statistically significant change in abundance (FDR < 0.05) between pre- and post-metformin treatment.^4^

Statistical analysis and graphs for in vitro and in vivo models were carried out using GraphPad Prism v6.0 (GraphPad). Methods used to estimate significance included one-way ANOVA and unpaired Student’s *t*-test. The latter was used unless otherwise described in the text. Standard error of the mean (SEM) was used to report variability unless otherwise indicated in the text. Based on our and others’ previous analyses of gene expression data, we estimated that a minimum of 20 cases with paired measurements at two time points were sufficient to observe expression changes of at least 1.7-fold in genes showing a coefficient of variation at each time point up to 50% with a significance level after multiple test correction of *p* = 0.05 (taking into account filtering of not expressed transcripts) and an 80% power. However, this estimate assumes uniformity of drug response and so double the number was desirable for higher significance and considering correlation with other markers.

### In vitro breast cancer cell line culture

All cell lines were purchased from the American Type Culture Collection within the past 2 years (LGC standards, UK). LGC standards routinely authenticate cell lines using short tandem repeat profiling. All cell lines were passaged for only a maximum of 3 months after resuscitation. All cell lines were tested for mycoplasma contamination prior to use. None of the cell lines used are listed in the database of commonly misidentified cell lines identified by the ICLAC. Human breast carcinoma cell lines MCF7, T47D, BT474, MDA-MB-231, MDA-MB-468 and MDA-MB-157 cells were all cultured in Dulbecco’s Modified Eagle Medium (DMEM) (Invitrogen) supplemented with 10% foetal bovine serum (FBS) and 100U/ml penicillin, and 100μg /ml streptomycin and cultured at 37 °C and in 5% CO_2_ in a humidified incubator. Metformin was purchased from Calbiochem, and etomoxir, AICAR and rotenone from Sigma–Aldrich.

### Gas chromatography analysis of TG and DG FA for in vitro samples

FA methyl esters (FAMEs) of adipocyte TGs were prepared and analysed by GC as described previously.^6^ FA concentrations were calculated relative to an internal standard (C15:0 TG), and the results expressed either as micrograms of FA per 4 × 10^6^ cells or as percentage of total TG. For DG:TG calculations, lipids were extracted as described except that the fractions were separated using thin layer chromatography rather than solid phase extraction columns and using a C15:0 DG internal standard.

### Use of stable isotopes in vitro to measure FA uptake and trace carbon contribution of DNL substrates

Fifty micromolar [U-^13^C]palmitate (CK Gas, Ibstock, UK) was added to MCF7 and MDA-MB-468 cells for 48 h. After harvesting cells, FAMEs were prepared. TGFAs were quantitated using GC and the isotopic enrichment of TG measured using gas chromatography-mass spectrometry (GC**–**MS) as previously described.^6^ Isotopically labelled DNL substrates were added to metformin-treated MCF7 cells to determine the effects of metformin on substrate contribution towards lipid synthesis. Twenty-five millimolar D-[U-^13^C]glucose, 4 mM [U-^13^C]glutamine and 5 mM [U-^13^C]lactate were used. FAMEs were prepared from harvested cells after 48 h of the indicated treatment and TGFAs were measured for amount of FA and isotopic enrichment of FA using GC and GC**–**MS, respectively. The contribution of each substrate towards DNL was calculated using quantitative mass spectral analysis as previously described.^7^ Briefly, we calculated the fraction of all 13 C carbon atoms in the product (e.g. palmitic acid). As the substrates [U-13C]glucose, [U-13C]glutamine and [U-13C]lactate were uniformly labelled, this represented the fraction of the product formed from the substrate being studied.

### Use of ^2^H_2_O to estimate DNL for in vitro samples

As previously described ^2^H_2_O was added to media to a concentration of 5%.^8^ Cells were then harvested after 48 h of the indicated treatment and TG and PL FAMEs prepared prior to GC and GC**–**MS analysis. Molar enrichment of each TGFA was measured and percentage synthesis calculated based on observed/theoretical molar enrichment. The amount of DNL-derived FA was then calculated from the total TGFA measured using GC analysis and expressed as µg FA/4 × 10^6^ cells or as percentage of total TGFA. PL analysis was carried out as per TGFA analysis except only measuring enrichment of palmitate due to the very low enrichment of other FAs in the PL fraction (likely due to lower levels of modification of PL DNL-derived palmitate).

### FAO measurements for in vitro samples

Cells were treated for 48 h with either 0, 1 or 2 mM metformin, with or without 100 µM etomoxir. FAO was then measured as previously described^9^ using 0.5 mM oleate and 0.5 µCi [^14^C]oleate (Perkin Elmer, UK) solution bound to bovine serum albumin in low (5 mM) glucose DMEM. Rate of oxidation was calculated as pmol CO_2_/h/4 × 10^6^ cells.

### Xenograft experiments and LD540 staining of frozen sections

All protocols were carried out under Home Office regulations and were approved by the University of Oxford Medical Sciences Division AWERB Committee. Six to eight-week-old female Balb-C nu/nu mice (Harlan) were injected into the mammary fat pad with 25 μl Matrigel (BD Bioscience) and 2.5 × 10^6^ MDA-MB-468 cells suspended in 25 μl serum free medium. Once greater than 50% of xenografts had reached a size greater than 150 mm^3^ half the mice were treated with metformin in the drinking water (750 mg/kg/day). Tumour volume was calculated from the formula V = L × W × H × π/6 (L = length, W = width, and H = height). Animals were not randomised but allocated to the metformin or no treatment arms with equal weighting for tumour size at baseline. The investigator was not blinded to the group allocation during the experiment. Once tumours reached 1.44 cm^3^ mice were sacrificed by cervical dislocation. Tumours were then snap frozen in liquid nitrogen and frozen sections cut and stained with LD540 and DAPI (Vectashield mounting medium with DAPI). A Zeiss Axio Observer Z1 inverted epifluorescence microscope was used to capture images of the (less necrotic) periphery of the tumour. The area staining for lipid with LD540 was then defined as a percentage of the area staining for DAPI using image analysis software (ImageJ).

### LD450 staining of intracellular lipid droplets in vitro and BODIPY uptake

MCF7 cells were seeded on sterile coverslips in 6-well plates overnight prior to treatment for 48 h with 0, 2 or 10 mM metformin treatment prior to removal and staining with LD540 and DAPI (Vectashield mounting medium with DAPI). Z-stack images were then created with laser scanning confocal microscopy (Zeiss LSM510 Meta). Control samples had oleic acid to a concentration of 250 μM added for 24 h prior to microscopy.

For BODIPY uptake measurements, cells were treated with the fluorescent palmitate analogue, 4,4-difluoro-5,7-dimethyl-4-bora-3a,4a-diaza-s-indacene-3-hexadecanoic acid (BODIPY^®^ FL C16, Invitrogen) for 15 min and then washed with phosphate buffered saline (PBS). After harvesting with trypsinisation the cells were fixed with 4% paraformaldehyde prior to re-suspension in PBS and then analysed using a FACSCalibur flow cytometer (BD Biosciences) and Cellquest software (BD Biosciences).

### In vitro cell growth assays

100,000 cells were plated on a 6-well dish and the following day the media replaced with DMEM media supplemented with metformin at a concentration of 0 (control) or 2 mM and/or 100μM etomoxir. The media was supplemented with or without 25 mM glucose, with or without 20 mM galactose and with 1 mM pyruvate and 4 mM glutamine. For the fatty acid supplementation experiment the media was supplemented with and without a mixture of fatty acid at a concentration of 0.2, 0.5 and 1 mM (oleate, palmitate and linoleate in a ratio of 45%, 30% and 25%, respectively). Cell number was counted at 96 h using an automated cell counter (Nexcelom). To maintain more consistent nutrient concentrations the media was replaced on a 24-hourly basis.

## Results

**Fig. 1:**
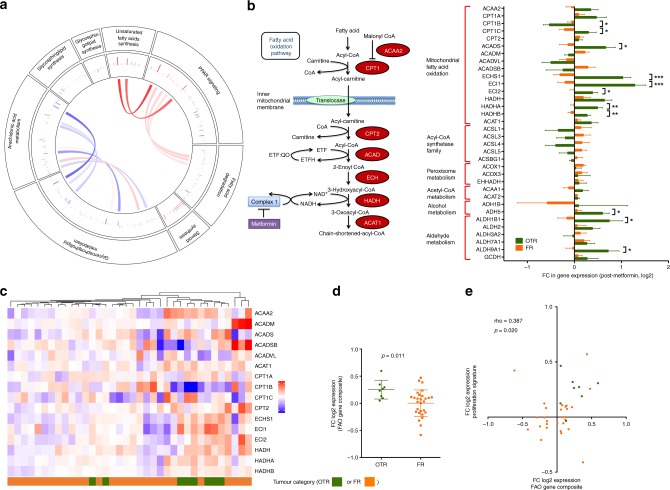
**a** Circos plot to show all lipid metabolism pathways in the KEGG database with significant changes in expression. The width of the outer circle shows the mean relative abundances for the secondary hierarchy. The bars in the innermost circle represent the mean relative abundances for genes encoding proteins within the individual pathways. The curved lines link genes that are shared among different pathways as indexed by KEGG Multiple lipid metabolism pathways that had significant changes in expression at the transcriptomic level. **b** Change in expression of genes involved in regulation of fatty acid degradation (all genes from KEGG:00071, does not include non-expressed genes in this KEGG pathway), unpaired *t*-test (*n* = 36). Data shown are mean ± SEM. *p < 0.05; **p < 0.01; ***p < 0.001. **c** Heatmap of differentially expressed genes from the fatty acid degradation pathway (KEGG:00071) but limited to key regulators of mitochondrial FAO. Each row represents a gene and each column represents a single patient (*n* = 36). Colours reflect the fold change for each gene post-metformin: Red = upregulation, Blue = downregulation. Samples were visually clustered using hierarchical clustering. OXPHOS transcriptional response group (OTR; eight patients) and FDG response group (FR; 28 patients) shown. **d** Scatter plot to show for the OXPHOS transcriptional response group (OTR) and FDG response group (FR) change in the composite FAO gene expression signature for the breast primary tumour (both post minus pre). Data shown are mean ± SEM, unpaired *t*-test. **e** Correlation between fold change in the proliferation metagene signature and change in the composite FAO gene expression signature. Spearman’s rank correlation coefficient and significance are shown.

We previously conducted a clinical pharmacodynamic study that integrated dynamic fluoro-deoxy-D-glucose positron emission tomography-computed tomography (FDG-PET-CT) imaging, transcriptomic and metabolomic analyses to characterise metformin’s effects on breast cancer metabolism (see Fig. S1A for study schema). This work identified two distinct metabolic responses to metformin in primary breast cancer, an oxidative phosphorylation (OXPHOS) transcriptional response (OTR) group for which there was an increase in OXPHOS gene transcription and an FDG response (FR) group with increased FDG uptake. This was linked to change in a validated transcriptomic proliferation metagene, which suggested that tumours in the OTR group were resistant to metformin.^4^ In that analysis our work focussed on changes in OXPHOS, glucose, glutamine and aspartate metabolism. However, it was striking that several lipid metabolism pathways had significant changes in expression at the transcriptomic level (Fig. 1a and Table S1) and that metabolomic profiling of primary breast tumour tissue showed a decrease in the level of propionylcarnitine, one of a family of short-chain acyl-carnitines that aid the shuttling of fatty acid oxidation degradation products out of mitochondria. Consistent decreases in short-chain acyl-carnitine levels have previously also been described in a clinical metabolomic study of metformin in ovarian cancer.^5^

Complex 1 is required to generate the cofactor NAD^+^ to catalyse the final dehydrogenation step of FAO and notably, in the transcriptomic analysis from our patient study, the fatty acid degradation pathway (KEGG:00071) was significantly upregulated (corrected hypergeometric *p*-value = 0.005, Fig. 1a). We then assessed the change in levels for all the expressed genes in this pathway for the OTR group vs. FR group. Of the 16 key genes directly involved in regulating mitochondrial FAO, eight genes had significant change in expression between the two groups. However, this was true for only three of the 18 genes that annotate to other fatty acid degradation processes such as aldehyde and peroxisomal fatty acid metabolism (Fig. 1b). Unsupervised hierarchical clustering of these mitochondrial FAO genes revealed that most of the tumours in the OTR group also clustered together in this analysis (8 patients in OTR group vs. Twenty-eight patients in the FR group) (Fig. 1c). Next, having determined the median fold change in expression for this set of mitochondrial FAO genes to provide a ‘FAO gene composite signature’, we observed a difference in the fold change in expression of this composite variable between the OTR and FR groups (Fig. 1d). Additionally, there was a positive correlation between the fold change in the FAO gene composite signature and change in a validated human breast cancer proliferation signature^10^ (Fig. 1e).

**Fig. 2:**
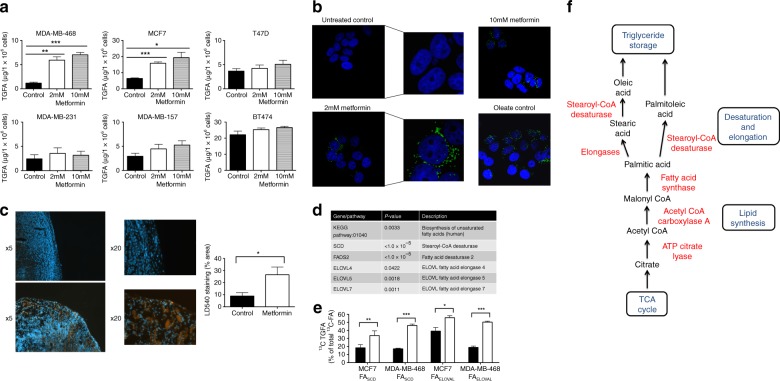
**a** For each cell line: cells were treated with either 2 or 10 mM metformin vs. control (no metformin) for 48 h and TGFAs measured using gas chromatography (GC). Data are expressed as mean total TGFA (µg/4 × 10^6^ cells) ± SEM (*n* = 3). **b** LD540 staining of lipid droplets (green) in MCF7 cells treated with 0, 2 and 10 mM metformin and control (MCF7 cells treated with 250 µM oleate). DAPI-stained nuclei are seen in blue. **c** LD540 staining of control and metformin-treated MDA-MB-468 xenografts. Image analysis quantification of the LD540 staining in the xenografts expressed as a percentage of the area staining for DAPI (*n* = 6, each group). **d** KEGG pathway and key genes relating to fatty acid modification with increased expression following metformin treatment in clinical study (all tumours; *n* = 36). **e** Effect of metformin on the SCD and ELOVL indices in MCF7 and MDA-MB-468 cells (*n* = 4–6). **f** Diagram to show initial desaturation and elongation steps for palmitic acid prior to storage triglyceride. Data shown are mean ± SEM. *p < 0.05; **p < 0.01; ***p < 0.001.

Next, we examined the effects of metformin in vitro in breast cancer cell lines. Lipid accumulation has been described as a consequence of reduced FAO inhibition.^11^ Using gas chromatography, the effect of metformin treatment on cellular triglyceride fatty acid (TGFA) content was assessed in three oestrogen/progesterone receptor (ER/PR) positive human breast cancer cell lines (MCF7 and T47D, BT474) and three ER/PR negative cell lines (MDA-MB-231, MDA-MB-468 and MDA-MB-157). Metformin led to dose-dependent TGFA accumulation in two of the six cell lines investigated: MCF7 and MDA-MB-468 (Fig. 2a).

Lipid droplets play a key role in the storage of TGFA thereby preventing lipotoxicity from free fatty acids. Staining with the lipophilic fluorescent dye, LD540, demonstrated an increase in lipid droplets in metformin-treated MCF7 cells (Fig. 2b). LD540 staining of metformin-treated MDA-MB-468 xenografts also demonstrated a marked increase in lipid accumulation compared with controls (percentage of DAPI staining area: 8.9 ± 2.8% vs. 26.6 ± 6.3% in control and metformin-treated xenografts respectively, *p* = 0.03) (Fig. 2c).

Monounsaturated fatty acids are preferred substrates for synthesis of TGFA prior to lipid droplet storage.^12^ Notably, the analysis of the transcriptomic data from our patient study for all tumours revealed that the biosynthesis of unsaturated fatty acids pathway (KEGG:01040) was significantly upregulated. In particular, the gene stearoyl-CoA desaturase (SCD), which catalyses the rate limiting step in the formation of monounsaturated fatty acids, had a highly significant increase in expression (Fig. 2d). Metformin treatment of both MCF7 and MDA-MB-468 cells led to an increase in desaturation of [U-^13^C]palmitate (percentage of TGFA in the desaturated fraction). There was also an increase in elongation of the exogenous fatty acids in the TG fraction (Fig. 2e, f).

**Fig. 3:**
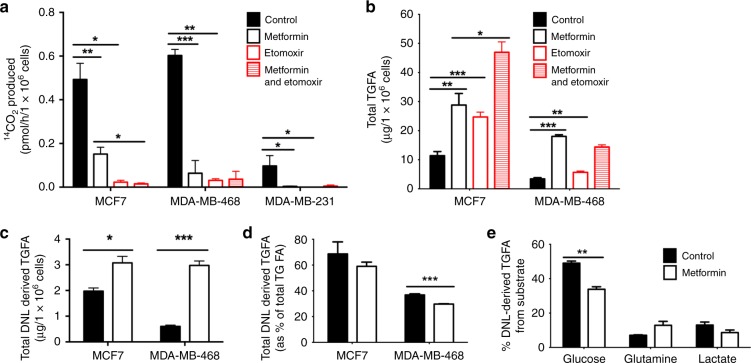
**a** Rate of oleate oxidation in MCF7, MDA-MB-468 and MDA-MB-231 cells after 48 h treatment with 2 mM metformin and/or 100 µM etomoxir (*n* = 3–4). **b** The effects of metformin (2 mM) and etomoxir (100 µM) on total TGFA accumulation in MCF7 and MDA-MB-468 cells (*n* = 4–6). **c** All six cell lines were treated with 2 mM metformin and/or 100μM etomoxir vs. control for 96 h in media supplemented with 25 mM glucose or with 20 mM galactose but no glucose and cell number measured (*n* = 6). **d** Accumulation of DNL-derived TGFA in MCF7 and MDA-MB-468 cells (*n* = 3). **e** Percentage of TGFA synthesised via DNL as estimated from ^2^H_2_O enrichment of fatty acid (*n* = 3). **f** Accumulation of TGFA in MCF7 cells derived from three different ^13^C-labelled substrates: glucose, glutamine and lactate (*n* = 3). Data shown are mean ± SEM. *P < 0.05; **P < 0.01; ***P < 0.001.

Direct measurement of FAO using the radiolabelled tracer [^14^C]oleate in MCF7 and MDA-MB-468 cells confirmed that metformin induced a marked reduction in FAO. Etomoxir (an inhibitor of mitochondrial fatty–acyl–carnitine transport and thus FAO) further reduced FAO compared to metformin alone in MCF7 cells highlighting that inhibition of FAO activity was incomplete after dosing at 2 mM metformin for this cell line. Metformin also reduced FAO in MDA-MB-231 cells but the rate of FAO in untreated cells was much lower for this cell line (Fig. 3a). Etomoxir alone led to TGFA accumulation in both MCF7 and MDA-MB-468 cells. Etomoxir alone led to TGFA accumulation in both MCF7 and MDA-MB-468 cells and in MCF7 cells, metformin in combination with etomoxir led to further TGFA accumulation compared with metformin or etomoxir alone (Fig. 3b).

Metformin reduced cell growth for MDA-MB-468 cells but not MCF7 and MDA-MB-231 cells in 25 mM glucose. Replacement of glucose with galactose forces cells to rely on mitochondrial respiration (OXPHOS and FAO).^13^ In glucose free media supplemented with 20 mM galactose, metformin treatment led to inhibition of cell proliferation in all three cell lines. Etomoxir alone inhibited proliferation of MCF7 cells and MDA-MB-231 cells under 25 mM glucose and glucose free/galactose supplemented conditions, respectively, suggestive of some reliance on FAO for tumour growth for these cell lines (Fig. 3c). The addition of a mixture of the three main dietary fatty acids (oleate, palmitate and linoleate) at 0.2, 0.5 or 1 mM did not lead to a significant additive effect on proliferation following treatment with 2 mM metformin when compared with no fatty acid (Fig. S1B), (one-way ANOVA).

De novo lipogenesis (DNL) is the synthesis of FA and incorporation into lipids, from non-lipid precursors. To determine the impact of metformin on DNL we measured the incorporation of deuterated water (^2^H_2_O) into lipids. Metformin treatment led to an accumulation of DNL-derived TGFA in metformin-treated MCF7 and MDA-MB-468 cells (Fig. 3d), although the proportion of DNL-derived TGFA within the total cellular TG pool in MDA-MB-468 cells decreased (Fig. 3e). Metformin reduced the proportion of TGFA derived from glucose in MCF7 cells (Fig. 3f).

**Fig. 4:**
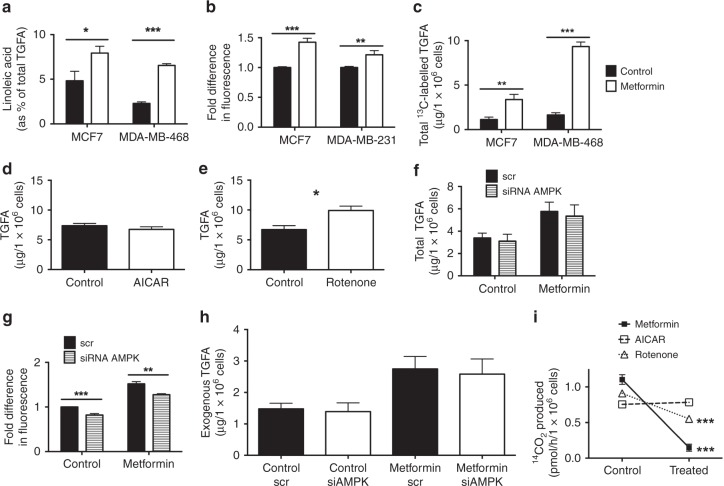
**a** Effect of 2 mM metformin on the levels of the essential fatty acid, linoleic acid, as a percentage of TG fatty acid in MCF7 and MDA-MB-468 cells (*n* = 3–7). **b** Effect of 2 mM metformin increased BODIPY C16 uptake in MCF7 and MDA-MB-231 cells (*n* = 3). **c** Effect of 2 mM metformin on ^13^C_16_-palmitate incorporation into TGFA in MCF7 cells (*n* = 5–6). **d** Effect of AICAR on triglyceride fatty acid accumulation in MCF7 cells (*n* = 3). **e** Effect of rotenone on triglyceride fatty acid accumulation in MCF7 cells (*n* = 3). **f** Effect of siRNA-mediated AMPK knockdown (siAMPK) on exogenous fatty acid accumulation after 48 h of treatment with 2 mM metformin in MCF7 cells (*n* = 3). **g** Effect of siAMPK on BODIPY-C16 uptake in control and 2 mM metformin-treated MCF7 cells (*n* = 3). **h** Effect of siAMPK on exogenous fatty acid accumulation after 48 h of treatment with 2 mM metformin in MCF7 cells (*n* = 3). **i** Effects of AICAR, rotenone and metformin on fatty acid oxidation in MCF7 cells (*n* = 3). Data shown are mean ± SEM. *P < 0.05; **P < 0.01; ***P < 0.001.

As the increase in DNL-derived FA did not account for all the observed increase in triglyceride accumulation in metformin-treated cells, we investigated metformin’s effects on cellular FA uptake. Essential fatty acids cannot be synthesised de novo and metformin increased the amount of the essential FA, linoleic acid, within MCF7 cells as a proportion of total TGFA (Fig. 4a). Metformin increased uptake of BODIPY**–**C16, a fluorescent palmitate analogue, in MCF7 cells to a greater extent than in MDA-MB-231 cells (fold difference in fluorescence vs. control cells 1.42 ± 0.07, *p* < 0.001 and, 1.21 ± 0.07, *p* = 0.008, in MCF7 and MDA-MB-231 cells, respectively) (Fig. 4b). Furthermore, metformin increased the accumulation of TGFA derived from exogenous [U-^13^C]palmitate in both metformin-sensitive MCF7 and MDA-MB-468 cells (Fig. 4c).

As metformin treatment of MCF7 cells led to a 2-fold reduction in the diacylglycerol (DG) to TG ratio (Fig. S1C) we assessed the effect of metformin on lipolysis. The total lipase activity was not decreased in metformin-treated MCF7 cells. Additionally, there was no change in ATGL activity or HSL activity (Fig. S1D).

Treatment of MCF7 cells with the activator of AMPK, AICAR, had no effect on TGFA accumulation (Fig. 4d). Conversely treatment with the complex 1 inhibitor, rotenone, led to an accumulation of TGFA (Fig. 4e). Using AMPK-targeted siRNA to the PRKAA1 and PRKAA2 subunits of AMPK, we achieved abrogation of phosphorylation of AMPK up to 72 h (Fig. S1E). Total TGFA increased with metformin treatment but was unaffected by siRNA-mediated AMPK silencing (Fig. 4f). AMPK knockdown in MCF7 cells did lead to a small reduction in BODIPY**–**C16 uptake both in control cells and metformin-treated cells (Fig. 4g). Despite this observation, metformin stimulated accumulation of exogenous TGFA (as measured by TGFA not enriched by ^2^H_2_O) was unaffected by AMPK knockdown, suggesting that FA uptake secondary to AMPK activation was not the driver for overall TGFA accumulation (Fig. 4h). Rotenone treatment significantly reduced [^14^C]oleate oxidation whilst there was a trend toward a modest increase for AICAR (Fig. 4i).

One possible explanation for the differential response between cell lines to metformin is expression of the organic cation transporters OCT1 and OCT2, which play a role in cellular drug influx.^14,15^ However, expression of these transporters was unexpectedly greatest in the resistant cell lines (MDA-MB-231 and BT474) (Fig. S1F). We also explored whether mitochondrial functional capacity reflected sensitivity to metformin. However, baseline oxygen consumption did not correlate with the degree of triglyceride accumulation in response to metformin across the six cell lines (Fig. S2). MDA-MB-468 was the most sensitive cell line to metformin’s effect on triglyceride accumulation, cell proliferation and mitochondrial respiration across the board (Fig. S3).

## Conclusions

Our clinical study suggests that, at least at the transcriptomic level, metformin modifies FAO in primary breast cancer at therapeutic dosing, resulting in adaptation of the fatty acid desaturation pathway. Consistent with this observation, levels of short-chain acyl-carnitines were decreased in the tumour tissue of metformin-treated patients. The positive correlation between changes in mitochondrial FAO gene transcription and expression of a proliferation signature suggests that mitochondrial FAO response to metformin treatment may link to therapeutic benefit.

Prior preclinical studies of metformin’s effects on FAO have focussed on mouse embryonic fibroblasts (MEFs), hepatocytes, hepatoma and cardiac and skeletal muscle cell models demonstrating increased FAO in an AMPK-dependent manner with a resultant decrease in cellular lipid content.^16–20^ However, in our cell line models of breast cancer, metformin inhibited FAO. We also observed an increase in total cellular TGFA, and we had hypothesised that this may partly be due to increased exogenous FA uptake. The expectation was that the main driver for this under conditions of ATP depletion would be AMPK activation^21^ and indeed AMPK knockdown resulted in reduced uptake of BODIPY in metformin-treated cells. However, knockdown of AMPK had no effect on accumulation of exogenous TGFA suggesting that any impact on TGFA accumulation by an AMPK induced increase in FA uptake is insignificant. Additionally, the accumulation of both DNL-derived TGFA and not just exogenous TGFA was suggestive of an alternative process other than just an increase in FA uptake. Both metformin and rotenone (another inhibitor of complex 1) decreased FAO (in contrast to AICAR) and hence we speculate that metformin’s effects on FAO are driven by the more direct effect on complex 1 rather than downstream modulation of AMPK activity. That metformin also had no effect on lipolysis suggests inhibition of FAO is the driver for TGFA accumulation and consistent with this hypothesis etomoxir mimicked metformin’s effect on TGFA accumulation.

The increase in the fraction of desaturated fatty acid observed in vitro is suggestive of FA processing prior to triglyceride storage in lipid droplets in order to prevent cellular toxicity from accumulation of saturated FAs.^12,22^ SCD has been found to have elevated expression at the mRNA and protein level in human tumours and SCD inhibitors have successfully blocked tumour growth in preclinical models. Recent work has demonstrated that SCD inhibition sensitised tumour cells to metformin and other compounds that target the electron transport chain^23^ and we propose that this therapeutic strategy should be further explored.

**Fig. 5:**
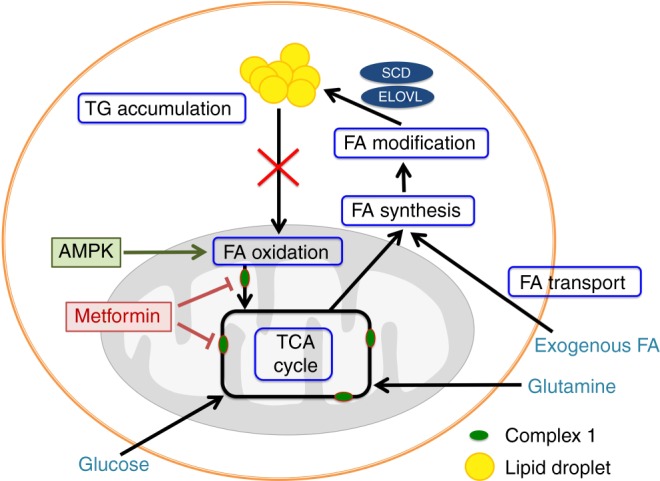
Model of effect of metformin on lipid metabolism on breast cancer cells. Triglyceride is synthesised from fatty acid derived from carbon sources including glucose and glutamine and also exogenous fatty acid, requiring modification by elongases and desaturases prior to storage in lipid droplets. Metformin inhibits complex 1 disrupting electron transfer required to catalyse the final dehydrogenation step of fatty acid oxidation resulting in the accumulation of triglyceride in lipid droplets. AMPK is known to be an activator of fatty acid oxidation and metformin’s predominant effects on lipid metabolism in breast cancer cells are via an AMPK-independent pathway and secondary to its more direct mitochondrial effect on Complex 1. FA fatty acid, TG triglyceride, TCA Cycle tricarboxylic acid cycle.

Our study showed relative consistent sensitivity between assays (TG accumulation, oxygen consumption and proliferation) for each cell line. We did not identify any specific molecular determinants for sensitivity and indeed demonstrated that low transporter protein expression did not match to assay response. However, a number of resistance mechanisms have been proposed for biguanides including mtDNA mutations in genes that encode for Complex 1.^24^ Mutations in LKB1, a kinase upstream of AMPK, have been reported in MDA-MB-231 cells and to mediate sensitivity to metformin.^25,26^ See Fig. 5 summarising our model with regard to the effect of metformin on lipid metabolism on breast cancer cells.

There are some limitations to this study. Most breast cancer gene expression proliferation signatures have been mapped to specific breast cancer subtypes and our signature was developed from a cohort of ER-positive and HER2-negative tumours (the most common subtype in our study) while this study was unselected for breast cancer subtype. Our patient cohort did not have an untreated control arm and it is possible that the passage of time (2 weeks) and repeat biopsy could alter gene expression analysis (or other assays), which in our study has not been controlled for.

In conclusion, these findings may have implications for repurposing metformin as an anti-cancer agent. Activation of FAO can rescue cancer cells under either drug induced stress (mTORC1 inhibitors) or loss of attachment from the extracellular matrix and FAO is a potential target for cancer therapy.^27^ Metformin has been shown to inhibit glucose oxidation and deplete ATP in cancer cells^28,29^ and the requirement of FAO for ATP production at times of energy stress has been shown in several different models.^27^ Metformin may selectively targets cancer stem cells^30–32^ and links have been made between active FAO and cancer stem cell maintenance and function.^33,34^ Epidemiological studies have suggested that metformin may decrease the risk of metastases in diabetic breast cancer patients receiving metformin^35^ and recently inhibition of FAO has been shown to markedly decrease metastasis and tumour growth in triple negative breast cancer xenograft models.^36,37^ Lastly, metformin’s modulation of cancer lipid metabolism may prove a useful biomarker of anti-cancer effect. Dynamic monitoring of metformin’s effects on FAO may define early response, for example, using the novel positron emission tomography tracer, ^18^F-fluoro-pivalic acid.^38^

## Supplementary information

Supplementary material

## Data Availability

Deposited RNASeq data: 10.17632/vhm5mtyk68.1
