# Supplementary material for: Transcriptomic analysis of human primary breast cancer identifies fatty acid oxidation as a target for metformin
Source: Br J Cancer. 2019 Dec 10;122(2):258–65. doi: 10.1038/s41416-019-0665-5 (PMC6986920; doi:10.1038/s41416-019-0665-5)
Supplement: Supplementary file 1 — Supplementary material [file 41416_2019_665_MOESM1_ESM.pdf]

Transcriptomic analysis of human primary breast cancer identifies fatty acid oxidation as a target for metformin

SUPPLEMENTAL FIGURES:

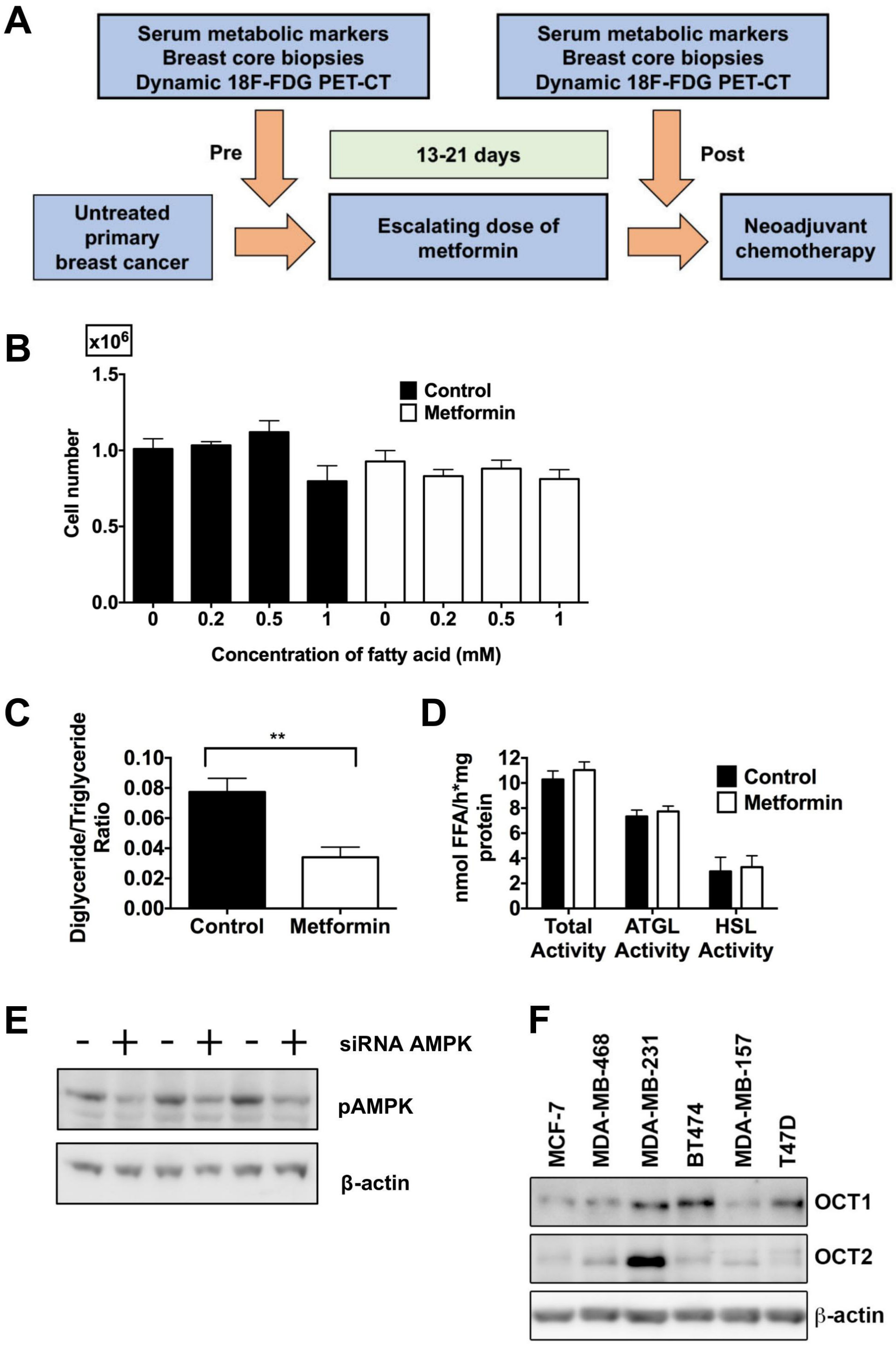

Fig S1. (**A**): Schema for pharmacodynamic clinical study (**B**): Effect of the addition of a mixture of fatty acids at a concentration of 0.2mM, 0.5mM and 1mM (oleate, palmitate and linoleate in a ratio of 45%, 30% and 25%, respectively) on proliferation of MCF7 cells with and without 2mM metformin (n=4). (**C**): Effect of metformin on the diglyceride to triglyceride ratio in MCF7 cells (n=5). (**D**): Effect of metformin on adipose triglyceride lipase (ATGL) and hormone sensitive lipase (HSL) activity (n=3). (**E**): Phosphorylation of AMPK in MCF7 cells at 72 hours after siRNA knockdown of the PRKAA1 and PRKAA2 subunits of AMPK. (**F**): Expression of OCT-1 and OCT-2 in all 6 cell lines. Data shown are mean ± SEM. *P < 0.05; **P < 0.01; ***P < 0.001.

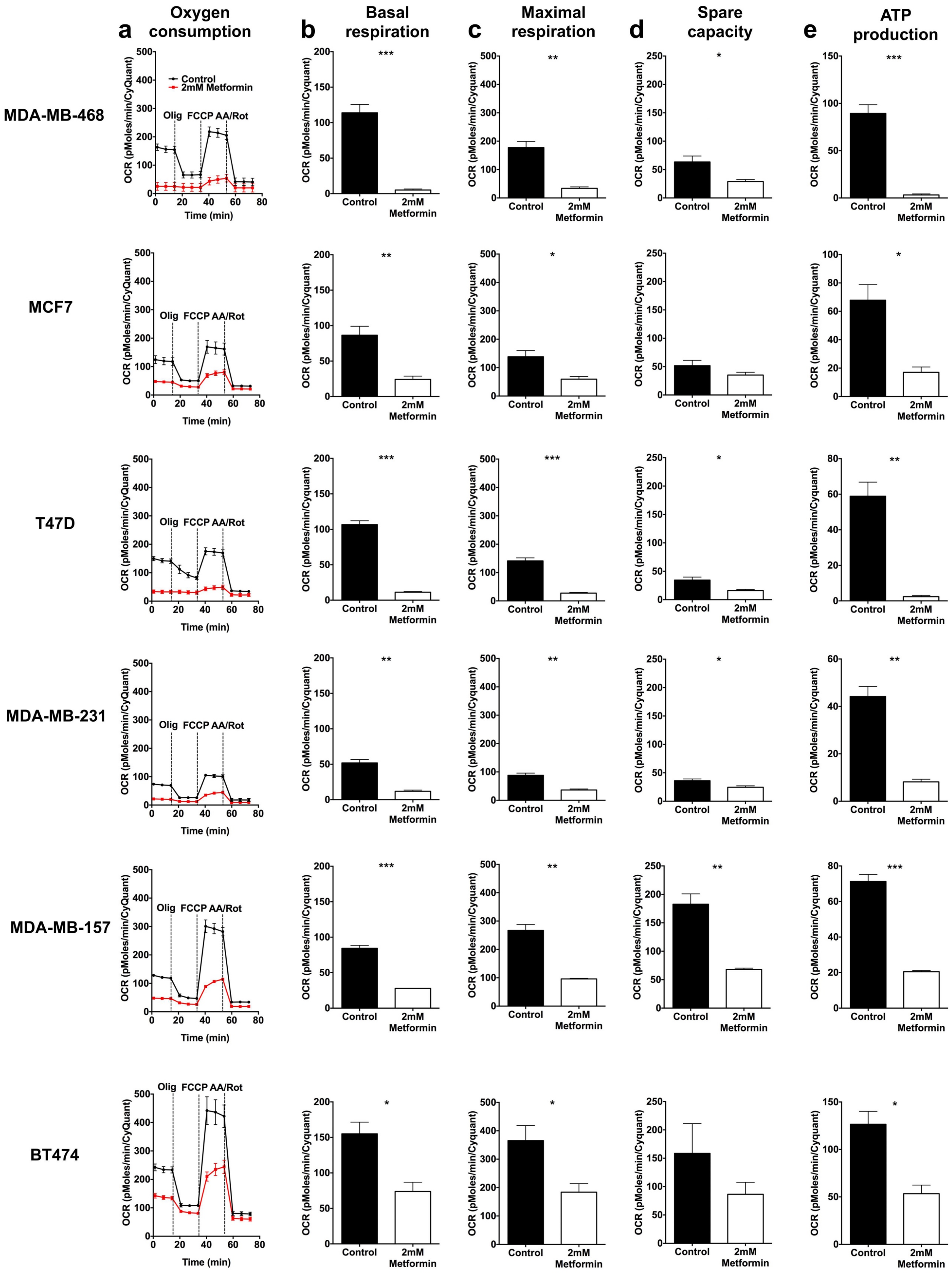

a
Oxygen consumption
b
Basal respiration
c
Maximal respiration
d
Spare capacity
e
ATP production
MDA-MB-468
MCF7
T47D
MDA-MB-231
MDA-MB-157
BT474
Control
2mM Metformin
Olig
FCCP
AA/Rot
OCR (pMoles/min/CyQuant)
OCR (pMoles/min/Cyquant)
Time (min)
Control
2mM Metformin

Fig S2. (**A**): Effect of 0 and 2mM metformin on oxygen consumption in all 6 cell lines. (**B-E**): Parameters of mitochondrial function determined from the same experiment. The oxygen consumption rate (OCR) prior to the addition of mitochondrial stressor agents is the basal respiration. The OCR measured after the injection of Mesoxalonitrile 4-trifluoromethoxyphenylhydrazone (FCCP) (which depolarizes the plasma and mitochondrial membranes uncoupling oxidative phosphorylation) is the maximal respiration. The spare capacity is the difference between maximal respiration and basal respiration. The addition of oligomycin blocks ATP synthase and any subsequent fall in the OCR correlates with ATP production. Data shown are mean ± SEM *p < 0.05; **p < 0.01; ***p < 0.001 (n = 3 for all experiments).

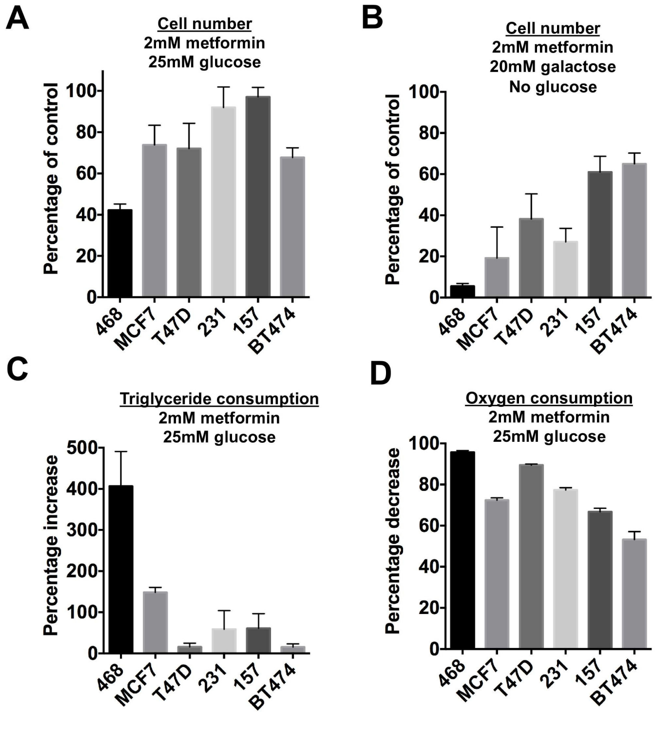

Fig S3. Percentage of cell number compared to control following 96 hours of treatment with 2mM metformin in media supplemented with (**A**): 25mM glucose (**B**): 20mM galactose but no glucose. (**C**): Percentage increase in triglyceride accumulation compared to control following 96 hours of treatment with 2mM metformin in media supplemented with 25mM glucose. (**D**): Percentage

decrease in oxygen consumption compared to control following 96 hours of treatment with 2mM metformin in media supplemented with 25mM glucose.

SUPPLEMENTAL TABLES:

**Pathway**	**KEGG ID**	**p-value***
Steroid biosynthesis	00100	<0.00001
Fatty acid degradation	00071	0.005
Biosynthesis of unsaturated fatty acids	01040	0.003
PPAR signaling pathway	03320	0.030
Glycosphingolipid synthesis	00601	0.041
Arachidonic acid metabolism	00590	0.003
Glycosphingolipid synthesis	00604	0.005
Glycerophospholipid metabolism	00564	0.019

Table S1. List of KEGG pathways linked to lipid metabolism that were significantly up or downregulated following metformin treatment (all tumours): Red = up-regulated, Blue = down-regulated.

* corrected Hypergeometric p-value.

SUPPLEMENTAL METHODS:

Measurements of total lipase activity

ATGL and HSL activity was measured as previously described by Das et al (Das et al., 2011) with minor modifications. 80µg of cell lysate was used for the assay. Positive controls of liver and white adipose

tissue were used. ATGL activity was determined by the addition of a hormone sensitive lipase (HSL) inhibitor and HSL activity was determined by subtraction of HSL inhibited lipase activity from total lipase activity.

AMPK knockdown

AMPKα1 (PRKAA1 On-TAplus SMART pool duplex, Dharmacon) and AMPKα2 (PRKAA2 On-TAplus SMART pool duplex, Dharmacon) were used for AMPK siRNA knockdown. siRNAs were transfected into MCF7 cells using Lipofectamine RNAiMax following the manufacturer's instructions (Invitrogen). On-TAplus non-targeting pool (Dharmacon) was used as a negative control.

Immunoblotting

Cell lysates were separated on 10% SDS-PAGE and transferred to a polyvinylidene difluoride membrane. The following primary antibodies were used pAMPKα(Thr172) and OCT-1 (#2535 and #8157, respectively, all Cell Signalling, USA), OCT-2 (ab198800, Abcam, UK) and β-actin (Sigma, UK).

Oxygen consumption assays

Oxygen consumption rate (OCR) was measured at 37°C using XF96 extracellular analyzer (Seahorse biosciences). Dependent upon the cell lines 17,500-20,000 cells were seeded per well on a 96 well plate and left to recover for 24 h prior to a media change with metformin added at varying concentrations as specified in the text. After metformin treatment overnight the cells were assayed using the XF96 extracellular flux analyser (Seahorse Bioscience). Prior to the assay, cells are prewashed 3 times with XF assay medium supplemented with 25 mM glucose, 4 mM glutamine, 1 mM pyruvate and metformin, and then equilibrated in XF assay medium at 37°C in a non-CO_2 incubator for 1 hour. All the medium is adjusted to pH 7.4 on the day of the assay. Three baseline measurements of OCR were taken before sequential injection of mitochondrial inhibitors. The respiratory chain inhibitors are ATP synthase complex inhibitor oligomycin, ATP synthesis uncoupler Carbonyl cyanide-*p*-trifluoromethoxyphenylhydrazone (FCCP), complex I inhibitor rotenone and complex III inhibitor antimycin A.
